# Sex-specific association of circulating Isthmin-1 with isolated post-challenge hyperglycemia

**DOI:** 10.3389/fendo.2024.1394190

**Published:** 2024-07-25

**Authors:** Jiahua Fan, Jialin He, Jiangyuan Zhu, Jialu Yang, Jingmeng Ju, Jingyi Huang, Zhihao Huang, Zhuoyu Zhang, Wenkang Li, Min Xia, Yan Liu

**Affiliations:** ^1^ Department of Clinical Nutrition, Guangzhou Chest Hospital, Guangzhou, Guangdong, China; ^2^ Guangdong Provincial Key Laboratory of Food, Nutrition and Health, Guangzhou, Guangdong, China; ^3^ Department of Nutrition, School of Public Health, Sun Yat-sen University, Guangzhou, Guangdong, China

**Keywords:** Isthmin-1, adipokine, isolated post-challenge hyperglycemia, diabetes, sexual dimorphism

## Abstract

**Introduction:**

To explore the distribution of Isthmin-1 (ISM1) level and its association with isolated post-challenge hyperglycemia (IPH).

**Methods:**

A total of 522 participants without a history of diabetes were invited to attend a standard 75g 2-h oral glucose tolerance test (OGTT), and 71 subjects were further invited for a 3-h oral minimal model test. Insulin sensitivity and β-cell function were evaluated using both HOMA and estimated from OGTT. Circulating ISM1 levels were determined by a commercially available ELISA kit.

**Results:**

A total of 76 (14.6%) participants were diagnosed as IPH, accounting for 61.3% of the newly diagnosed diabetes. ISM1 levels were significantly higher in men than in women (1.74 ng/mL *versus* 0.88 ng/mL). The inverse correlation between ISM1 and β-cell function and IPH was only significant in men. After multivariate adjustment, per unit increment in ISM1 was associated with 0.68-fold (95% CI: 0.49-0.90) reduced odds ratio (OR) of IPH in men. Compared to men with the lowest ISM1 levels, the adjusted OR of IPH with the highest ISM1 levels decreased by 73% (95% CI: 0.11-0.61). Moreover, incorporation of ISM1 into the New Chinese Diabetes Risk Score (NCDRS) model yielded a substantial improvement in net reclassification improvement of 58% (95% CI: 27%-89%) and integrated discrimination improvement of 6.4% (95% CI: 2.7%-10.2%) for IPH.

**Conclusions:**

ISM1 was significantly and independently associated with IPH, and serves as a feasible biomarker for the early identification of men with high risk of IPH.

## Graphical Abstract

ISM1 was significantly and independently associated with IPH, and might be serves as a feasible biomarker for the early identification of men with high risk of IPH.

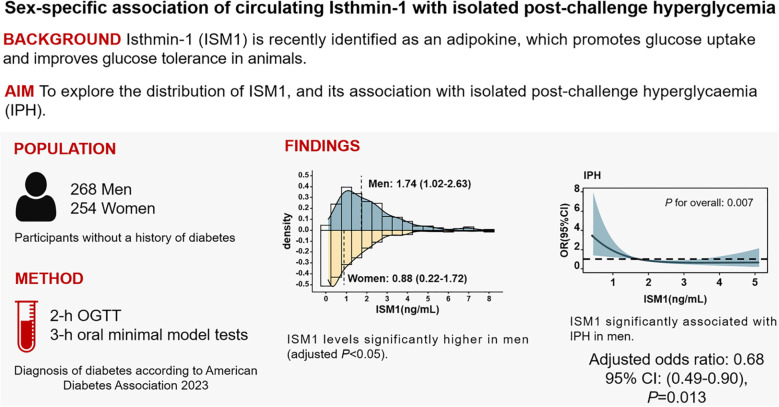

## Highlights

- Isthmin-1 (ISM1) is a newly identified adipokine which promotes glucose uptake and improves glucose tolerance in murine models. However, its distribution and association with glycemic control in general population remain largely unknown.
- To explore the sex-specific distribution of ISM1 and its association with post-challenge hyperglycemia.
- Circulating ISM1 is substantially higher in men than women. Significant association of ISM1 with post-challenge hyperglycemia exists only in men.
- Circulating ISM1 serves as a potential biomarker for post-challenge hyperglycemia in men.

## Introduction

Isolated post-challenge hyperglycemia (IPH), typically defined as 2-h glucose level higher than 11.1 mol/L with normal fasting glucose, is an established feature of diabetes and has been epidemiologically associated with worsening cardiometabolic health (1–3) and increased risk of mortality and morbidity (4–6). It is estimated that 46.6% of adults with undiagnosed diabetes have IPH in China (7), and the prevalence of IPH has been reported to account for up to half of the newly diagnosed diabetes in Asian populations (8). Timely pharmacological or lifestyle interventions to decrease the long-term risk in individuals with IPH is therefore mandatory. To date, however, IPH can only be detected by 2-h OGTT, which is time-consuming, inconvenient and not generally included in regular physical examinations, thus leaving a large proportion of patients with IPH undiagnosed and untreated. Clearly, considering the high and increasing prevalence of diabetes worldwide (9), identifying novel biomarkers for easy and early identification of subjects at high risk of IPH is of significance.

Isthmin-1 (ISM1), was first identified as a gene expressed in the Xenopus midbrain-hindbrain organizer called isthmus, with a role in early brain development (10). Recently, it was found to be a secreted polypeptide hormone that regulates adipose tissue glucose uptake (11), which plays an essential role in the maintenance of post-challenge glucose homeostasis (11). Ablation of ISM1 impaired both basal and insulin-stimulated glucose uptake by adipocytes, whereas, therapeutic administration of recombinant ISM1 improved diabetes in diet-induced obese mice (11). Moreover, despite a small sample size, ISM1 was proven to be detectable in the circulation and was reported to be lower in patients with type 2 diabetes than in those with normoglycemic control (12). However, as a newly identified adipokine, clinical investigation of circulating ISM1 in the general population is sparse and its association with IPH remains largely unknown.

In this study, we assessed the distribution of circulating ISM1 in a deeply phenotyped cohort of community residents and determined its association with IPH.

## Research design and methods

### Study population

This study was approved by the Ethics Committee of the School of Public Health, Sun Yat-sen University (2019-127) and was conducted in accordance with the principles of the Declaration of Helsinki. Written informed consent was obtained from each individual. Local residents, who had lived in Dongguan City, Guangdong, China for over 5 years were invited for diabetes screening through flyers and advertisements. The detailed inclusion and exclusion criteria were as follows:

*Inclusion criteria*: 1) aged 30-65 years old; 2) stable body weight (<10% of body weight change) for three months before the study; and 3) absence of diagnosed diabetes.

*Exclusion criteria*: 1) history of malignant tumor, severe liver or kidney disease, chronic hepatitis, thyroid disorders, autoimmune diseases, cardiovascular or cerebrovascular diseases; and 2) women during pregnancy or lactation.

A total of 535 participants were consecutively recruited, and 13 participants with outlier ISM1, defined as an extreme value of the upper 2.5% of the distribution (13) were excluded. Finally, a total of 522 participants were included in the current analysis.

### Collection and assessment of covariates

Demographic characteristics, medication, and lifestyles were collected through structured questionnaires by trained staffs. Current smokers were defined as those who had smoked at least one cigarette per day for more than 6 months (14). Current drinking was defined as taking any alcoholic beverage at least once a week in the past 6 months (14). Physical activity was assessed by the short form of the International Physical Activity Questionnaire (IPAQ). Participants were categorized into low, moderate and high levels according to the guidelines for data processing and analysis of the IPAQ and subjects with moderate or higher intensity activity was defined as having an exercise behavior (14).

Physical examinations were carried out by trained personnel. Blood pressure was measured on the right upper arm in the sitting position after at least 10-15 minutes of rest using a validated digital automatic analyzer (Omron HEM-7136). Hypertension was defined as systolic blood pressure (SBP) ≥140 mmHg, diastolic blood pressure (DBP) ≥90 mmHg, self-reported hypertension previously diagnosed by physician or current use of antihypertensive medications (15, 16). Overweight was defined as BMI ≥25 kg/m^2^, and central obesity was defined as waist circumference ≥80 cm for women and 94 cm for men (17). Metabolic syndrome was defined according to the National Cholesterol Education Programme Adult Treatment Panel III (NCEP ATP III) criteria (18).

### Biochemical measurements

Venous blood was taken after overnight fasting for 10-12 hours and subjected to laboratory analysis at local clinical laboratory. Glucose (fasting, 1-h and 2-hpost 75g glucose challenge), triglyceride (TG), total cholesterol (TC), HDL cholesterol (HDL-c), LDL cholesterol (LDL-c) and serum creatinine (Cr) concentrations were measured using an autoanalyzer (BS-800 Biochemical Autoanalyzer; Mindray; China). Estimate glomerular filtration rate (eGFR) was calculated using the Chronic Kidney Disease Epidemiology Collaboration equation (CKD-EPI) (19). Glycated hemoglobin (HbA1c) was measured by a designated high-performance liquid chromatography method (HA-8180, Arkray, Shiga, Japan). Insulin and C-peptide levels were determined with commercial ELISA kits (Mercodia, Uppsala, Sweden).

### Ascertainment of glucose metabolism

**Figure 1:**
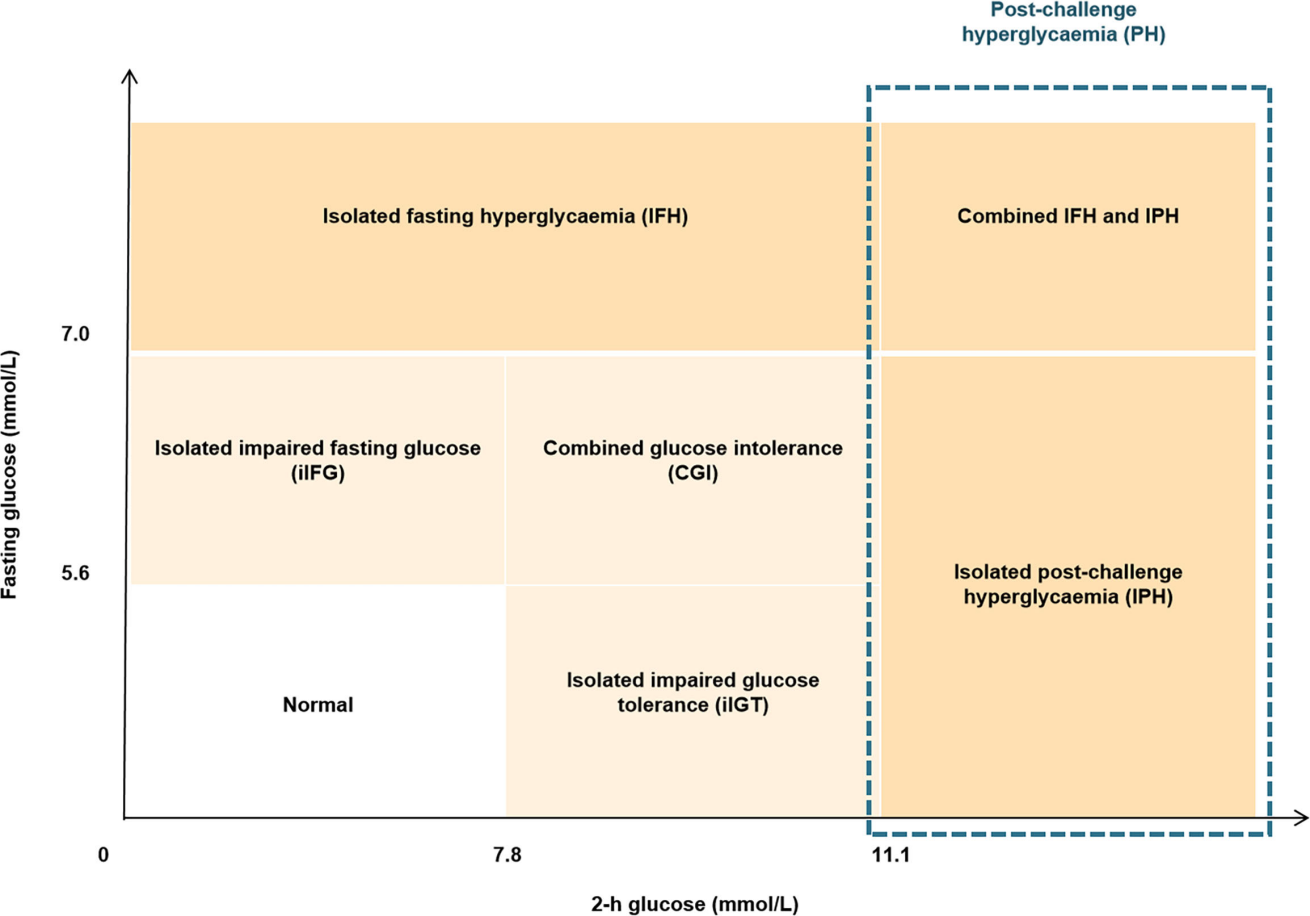
Diagram showing the definition of glycemic status. The definition and diagnostic criteria of different glycemic status was based on 2-h OGTT according to the guideline from American Diabetes Association.

All eligible participants were asked to fast overnight and were required to attend the 2-h standard oral glucose tolerance test (OGTT), with blood sampling at 0, 60 min and 120 min after oral loading of 75 g glucose. Among them, 71 subjects (38 men and 33 women) further participated in the 3-h oral minimal model tests with blood sampling at 0, 10, 20, 30, 60, 90, 120, and 180 min after a 75-g oral glucose challenge (20, 21). According to the American Diabetes Association (ADA) thresholds of glycemic categories (22), newly diagnosed diabetes was defined as fasting blood glucose (FBG) ≥ 7.0 mmol/L (126 mg/dL) or 2-h glucose ≥ 11.1 mmol/L (200 mg/dL); post-challenge hyperglycemia (PH) was defined as 2-h glucose ≥11.1 mmol/L (200 mg/dL); IPH was defined as FPG < 7.0 mmol/L (<126 mg/dL) and 2-h glucose ≥11.1 mmol/L (200 mg/dL); prediabetes was defined FBG of 5.6-6.9 mmol/L (100-125 mg/dL) or 2-h glucose of 7.8-11.0 mmol/L (140-199 mg/dL); impaired glucose tolerance (IGT) was defined as 2-h glucose of 7.8-11.0 mmol/L (140-199 mg/dL); isolated impaired glucose tolerance (iIGT) was defined as FBG < 5.6 mmol/L (< 100 mg/dL) and 2-h glucose of 7.8-11.0 mmol/L (140-199 mg/dL). Detailed definition and classification criteria of glucose status based on the 2-h OGTT are shown in **Figure 1**.

### Determination of ISM1

Fasting serum ISM1 levels were determined by a commercial ELISA kit (Biomatlk, Canada) according to the manufacturer’s instructions and compared with purified human ISM1 standards. The mean intra-assay and inter-assay coefficients of variation were 2.9-8.5% and 4.3-14.9%, respectively.

### Measurements of insulin sensitivity and β-cell function

The area under the curve (AUC) of glucose (AUC_glu_) and insulin (AUC_ins_) over the 2 hours was calculated with the trapezoidal method (23). Insulin sensitivity was estimated by the following indices: 1) the homeostatic model assessment of insulin resistance (HOMA-IR) = FBG (mmol/L) × fasting insulin (mU/L)/22.5; 2) TyG index = log [TG (mg/dL) × FBG (mg/dL)/2] (24) and 3) Matsuda index = 10000/√[Glu_0_ (mg/dL) × Ins_0_ ((mU/L)] × [Glu_mean_ (mg/dL) × Ins_mean_ ((mU/L)] (25). β-Cell function was evaluated by the following 5 indices: 1) HOMA-β = fasting insulin (mU/L) × 20/[FBG (mmol/L) - 3.5]; 2) Stumvoll first phase insulin secretion = 2,032 + 4.681 × Ins_0_ (pmmol/L) - 135.0 × Glu_120_ (mmol/L) + 0.995 × Ins_120_ + 27.99 × BMI - 269.1 × Glu_0_ (mmol/L) (26); 3) Stumvoll second phase insulin secretion = 277 + 0.800 × Ins_0_ (pmmol/L) - 42.79 × Glu_120_ (mmol/L) + 0.321 × Ins_120_ (pmmol/L) + 5.338 × BMI; 4) The insulinogenic index (IGI_60_) = [Ins_60_ (mU/L) - Ins_0_ (mU/L)]/[Glu_60_ (mmol/L) - Glu_0_ (mmol/L)] (27); and 5) The oral disposition index (DI) was evaluated as AUC_ins_/AUC_glu_ multiple by Matsuda index (27). Ins_0_, Ins_60_, Ins_120_, Glu_0,_ Glu_60_ and Glu_120_ were insulin and glucose during 2-h OGTT at 0, 60 and 120 min, respectively, and the units of insulin and glucose were shown in the above formula. Glu_mean_ and Ins_mean_ referred to the mean values of glucose and insulin at 0 and 120 min in 2-h OGTT.

### Oral minimal model-derived measurements

During the 3-h OGTT, glucose, insulin and C-peptide concentrations were measured in mmol/L, mU/L and nmol/L, respectively. The insulinogenic index (IGI) = (Ins_30_ - Ins_0_)/(Glu_30_ - Glu_0_) and C-peptide index (CPI) = (Cpep_30_ - Cpep_0_)/(Glu_30_ - Glu_0_). Oral minimal model-derived measurements, including the insulin sensitivity index (SI), dynamic insulin secretion (PhiD), static insulin secretion (PhiS) and total insulin secretion (PhiT), were calculated. Insulin sensitivity was estimated by the insulin/glucose minimal model, insulin secretion measures were derived from the glucose/C-peptide minimal model with SAAM II 2.3 software (The Epsilon Group, Charlottesville, VA), and the glucose derivatives were determined by MATLAB R2016a software (MathWorks, Natick, MA), as previously described (28).

### Statistical analysis

The normal distribution assumption was tested with the Shapiro-Wilk test. Data are presented as the mean ± SD for normally distributed variables and median with interquartile range for non-normally distributed parameters. Categorical variables were compared by χ^2^ test, and continuous variables were compared with independent Student’s *t* test or the Mann-Whitney U test as appropriate. To assess sex differences in ISM1, a least-squares regression model adjusted for age, history of hypertension, BMI, waist circumference, TG, HDL-c, eGFR and HOMA-IR was performed. In addition, differences in ISM1 levels according to different metabolic statuses were also determined by the least-squares regression model adjusted for age, and the box plots were based on the residuals from ordinary regression models of each response on age. Associations of ISM1 with various metabolic parameters, and indices related to insulin sensitivity and β-cell function were determined by age-adjusted partial Spearman correlation analysis. Linear regression models were performed to analyze the association of ISM1 with oral minimal model-derived measurements.

To evaluate the independent association of ISM1 with IPH in men and women separately, both linear and non-linear associations of ISM1 and glycemic status were first examined with restricted cubic spline (RCS) with 3 knots at the 10^th^, 50^th^ and 90^th^ centiles fitted in the multivariable logistic regression model. Age, history of hypertension, TG, HDL-c, eGFR, waist circumference and HOMA-IR were included in the RCS model as covariates. Furthermore, as a categorical variable, the independent associations of ISM1 with odds ratios (ORs) of IPH in men and women were determined with multivariable logistic regression model adjusted for potential confounders as specified below. Model 1: adjusted for age, history of hypertension, TG, HDL-c and eGFR. Model 2: Model 1 plus waist circumference. Model 3: Model 2 plus HOMA-IR. Moreover, to assess the additive value of ISM1 to the New Chinese Diabetes Risk Score (NCDRS), an established model for screening undiagnosed diabetes in Chinese individuals (29), the C-statistic, continuous net reclassification improvement (NRI) (30) and the integrated discrimination improvement (IDI) were employed. The likelihood ratio test was used to compare these models, and the calibration of these models was evaluated by the Hosmer-Lemeshow statistic. The confidence intervals (CIs) for the NRI values were computed using 1000 bootstrap samples.

Additionally, to enhance the robustness of our findings, several sensitivity analyses were performed. First, we restricted the analysis to participants who had complete data on 2-h OGTT tests and HbA1c results (n=428), where IPH was determined on both the OGTT test and HbA1c following the ADA guidelines (22). Second, ISM1 outliers were included in the analysis. Third, we expanded subjects with IPH into subjects with PH, defined as individuals with 2-h post-challenge hyperglycemia regardless of the level of fasting glucose. Finally, the E-value methodology was employed to assess the risk of residual confounding (31). We computed an E-value for the primary analysis using an online platform (32). Two-sided *P* values <0.05 were considered statistically significant and all statistical analyses were performed with R software, version 4.1.2.

## Results

### Sex-specific distribution of ISM1

The demographic and clinical characteristics of the study participants stratified by sex are presented in **Table 1**. Overall, the mean age was 47 years, and 48.7% were women. A total of 76 (14.6%) individuals were classified as IPH, accounting for 61.3% of the newly diagnosed diabetes in our cohort. Although there was no obvious difference in age, men were more likely to have unhealthy lifestyles, such as smoking and drinking, a higher prevalence of metabolic disorders, including diabetes, hypertension, dyslipidemia and metabolic syndrome, and worse metabolic profiles in terms of waist circumference, blood pressure and eGFR (*P <*0.05). Moreover, compared to women, both insulin sensitivity and β-cell function were markedly worse in men (**Table 1**).

**Table 1:** Participant characteristics stratified by sex.

	Total	Women	Men	*P* value
	(n = 522)	(n = 254)	(n = 268)	
ISM1, ng/mL	1.28 (0.61-2.30)	0.88 (0.22-1.72)	1.74 (1.02-2.63)	< 0.001
Demographics and laboratory variables				
Age, years	47.11 ± 7.22	47.63 ± 7.23	46.62 ± 7.20	0.109
BMI, kg/m^2^	25.18 ± 3.32	24.41 ± 3.33	25.92 ± 3.14	< 0.001
Waist circumference, cm	87.29 ± 9.57	82.89 ± 8.81	91.45 ± 8.34	< 0.001
SBP, mmHg	124 (113.5-135)	120 (109-133)	127 (118-138)	< 0.001
DBP, mmHg	84 (77-90.5)	80 (75-87)	86 (81-92)	< 0.001
TG, mmol/L	1.60 (1.07-2.43)	1.28 (0.93-1.92)	2.01 (1.33-2.85)	< 0.001
TC, mmol/L	5.16 (4.64-5.94)	5.20 (4.72-6.03)	5.09 (4.54-5.89)	0.075
HDL-c, mmol/L	1.25 (1.07-1.5)	1.43 (1.21-1.66)	1.12 (0.99-1.28)	< 0.001
LDL-c, mmol/L	3.30 ± 0.80	3.28 ± 0.83	3.32 ± 0.78	0.578
eGFR, mL/min/1.72m^2^	95.95 (82.4-107.57)	99.29 (87.16-109.76)	93.45 (77.7-105.29)	< 0.001
Fasting glucose, mmol/L	5.50 (5.06-6.00)	5.40 (5.00-5.81)	5.52 (5.08-6.10)	0.057
2-h glucose, mmol/L	8.12 (6.70-10.69)	7.80 (6.68-9.50)	8.50 (6.70-11.56)	0.062
Fasting insulin, mU/L	7.53 (5.25-11.59)	6.85 (4.92-10.7)	8.08 (5.67-11.95)	0.012
2-h insulin, mU/L	55.88 (32.58-93.88)	52.62 (33.50-93.13)	57.11 (31.43-94.18)	0.894
HbA1c, % ^#^	5.8 (5.5-6.1)	5.7 (5.4-6.0)	5.8 (5.6-6.2)	0.026
HbA1c, mmol/mol ^#^	40 (37-43)	39 (36-42)	40 (38-44)	0.026
AUC_glu_, mmol/L×h	17.26 (14.51-21.08)	16.42 (13.76-19.89)	18.18 (15.58-21.96)	< 0.001
AUC_ins_, mU/L×h	101.19 (68.55-162.72)	98.68 (66.75-148.90)	106.44 (69.89-172.39)	0.11
Insulin sensitivity				
HOMA-IR	1.87 (1.29-2.89)	1.65 (1.14-2.76)	2.00 (1.39-3.07)	0.008
TyG index	3.86 ± 0.29	3.76 ± 0.26	3.95 ± 0.29	< 0.001
Matsuda index	4.02 (2.63-6.05)	4.44 (2.79-6.77)	3.77 (2.50-5.33)	0.003
β-cell function				
HOMA-β	77.39 (49.94-119.46)	68.86 (48.66-116.51)	83.94 (53.2-124.95)	0.056
Stumvoll 1	755.93 (429.48-1027.01)	759.77 (497.1-987.82)	738.41 (281.17-1081.03)	0.784
Stumvoll 2	216.56 (149.68-281.45)	217.14 (154.02-268.63)	214.72 (140.29-286.11)	0.809
IGI60	13.17 (7.31-22.96)	14.12 (8.25-25.03)	12.24 (6.63-19.57)	0.016
DI	25.46 (17.19-33.43)	27.56 (19.78-35.87)	23.24 (15.27-31.32)	< 0.001
Lifestyle and Comorbidities, n (%)				
Current smoker	95 (18.2)	1 (0.4)	94 (35.1)	< 0.001
Current drinker	60 (11.5)	3 (1.2)	57 (21.3)	< 0.001
Physical activity	177 (33.9)	88 (34.6)	89 (33.2)	0.729
Hypertension	204 (39.1)	75 (29.5)	129 (48.1)	< 0.001
Dyslipidemia	263 (50.4)	95 (37.4)	168 (62.7)	< 0.001
Metabolic syndrome	188 (36.0)	76 (29.9)	112 (41.8)	0.006
Prediabetes	220 (42.1)	113 (44.5)	107 (39.9)	0.291
Impaired glucose tolerance	166 (31.8)	82 (32.3)	84 (31.3)	0.818
Isolated impaired glucose tolerance	94 (18.0)	52 (20.5)	42 (15.7)	0.154
Diabetes	124 (23.8)	49 (19.3)	75 (28.0)	0.026
Post-challenge hyperglycemia	120 (23.0)	47 (18.5)	73 (27.2)	0.023
Isolated post-challenge hyperglycemia	76 (14.6)	29 (11.4)	47 (17.5)	0.063

Data were shown as mean ± SD, median (lower quartile, upper quartile), or n and percent (%). P value indicated the difference between men and women. ^#^The total number was 428, with 211 women and 217 men.

**Figure 2:**
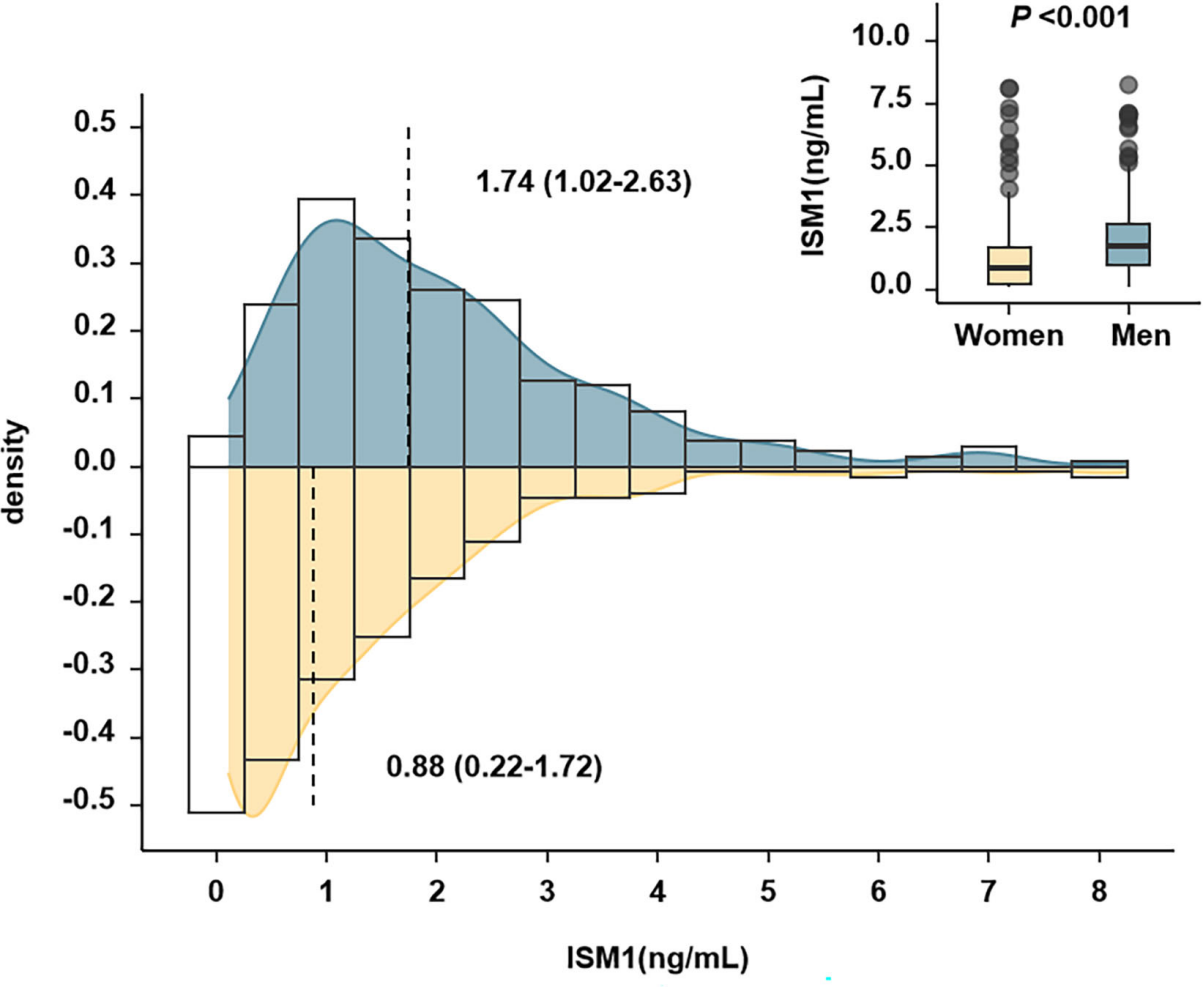
Distribution of Isthmin-1 according to sex. The *P* value indicated comparison between men and women after adjustment for age, history of hypertension, TG, HDL-c, eGFR, BMI, waist circumference and HOMA-IR. There was a significant difference in the distribution of circulating ISM1 by sex, with a median of 1.74 (1.02-2.63) ng/mL in men and 0.88 (0.22-1.72) ng/mL in women (*P <*0.001).

Of note, there was a significant difference in the distribution of circulating ISM1 by sex, with a median of 1.74 (1.02-2.63) ng/mL in men and 0.88 (0.22-1.72) ng/mL in women (*P <*0.001). The higher level of ISM1 in men remained significant even after adjustment for age, history of hypertension, TG, HDL-c, eGFR, BMI, waist circumference and HOMA-IR (*P <*0.05, **Figure 2**).

### Sex-specific association of ISM1 with metabolic parameters, insulin sensitivity and β-cell function

**Figure 3:**
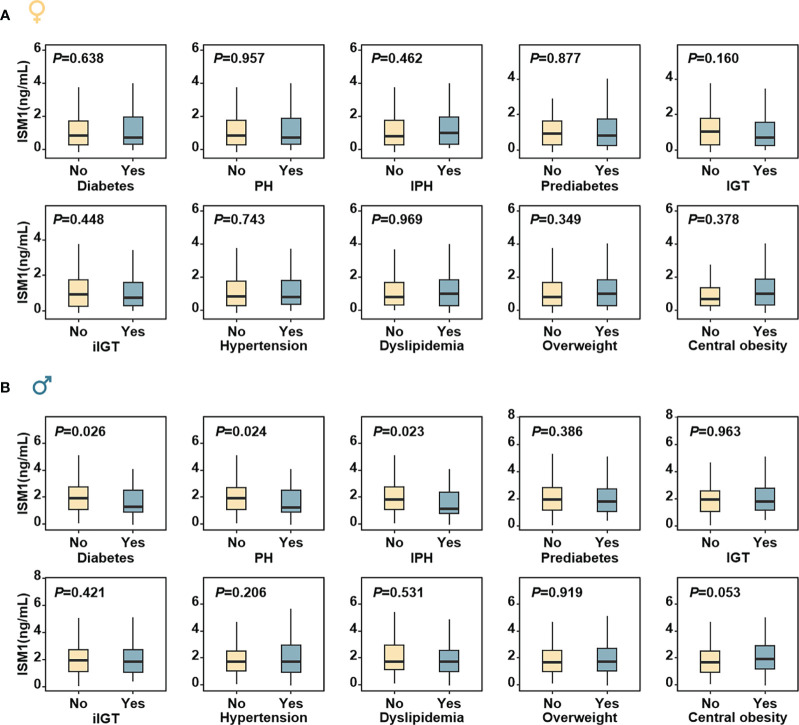
Distribution of ISM1 levels according to metabolic status in men and women. The box plots were based on the residuals from ordinary regression models of each response on age in **(A)** women and **(B)** men, respectively, according to different metabolic statuses. The P values were for comparisons between different metabolic statuses (yes and no) and adjusted for age. PH, post-challenge hyperglycemia; IPH, isolated post-challenge hyperglycemia; IGT, impaired glucose tolerance; iIGT, isolated impaired glucose tolerance.

In women, no significant difference in metabolic parameters, except for slightly decreased eGFR and improved HOMA-β was found with increasing tertiles of ISM1 (*P >*0.05, **Supplementary Table S1**). In contrast, in men, stumvoll 1 and stumvoll 2, two widely used measurements for insulin secretion capacity (26), were substantially impaired in men in the lowest tertile of ISM1. Consistently, the prevalence of diabetes, PH and IPH were also found to be remarkably higher in men with the lowest tertile of ISM1 expression (*P <*0.05, **Supplementary Table S2**). In a similar fashion, no obvious difference in ISM1 levels between females with different glycemic statuses or metabolic comorbidities was found (all *P* values >0.05, **Figure 3A**), while significantly lower ISM1s were observed in males with diabetes, PH and IPH (*P <*0.05, **Figure 3B**).

**Figure 4:**
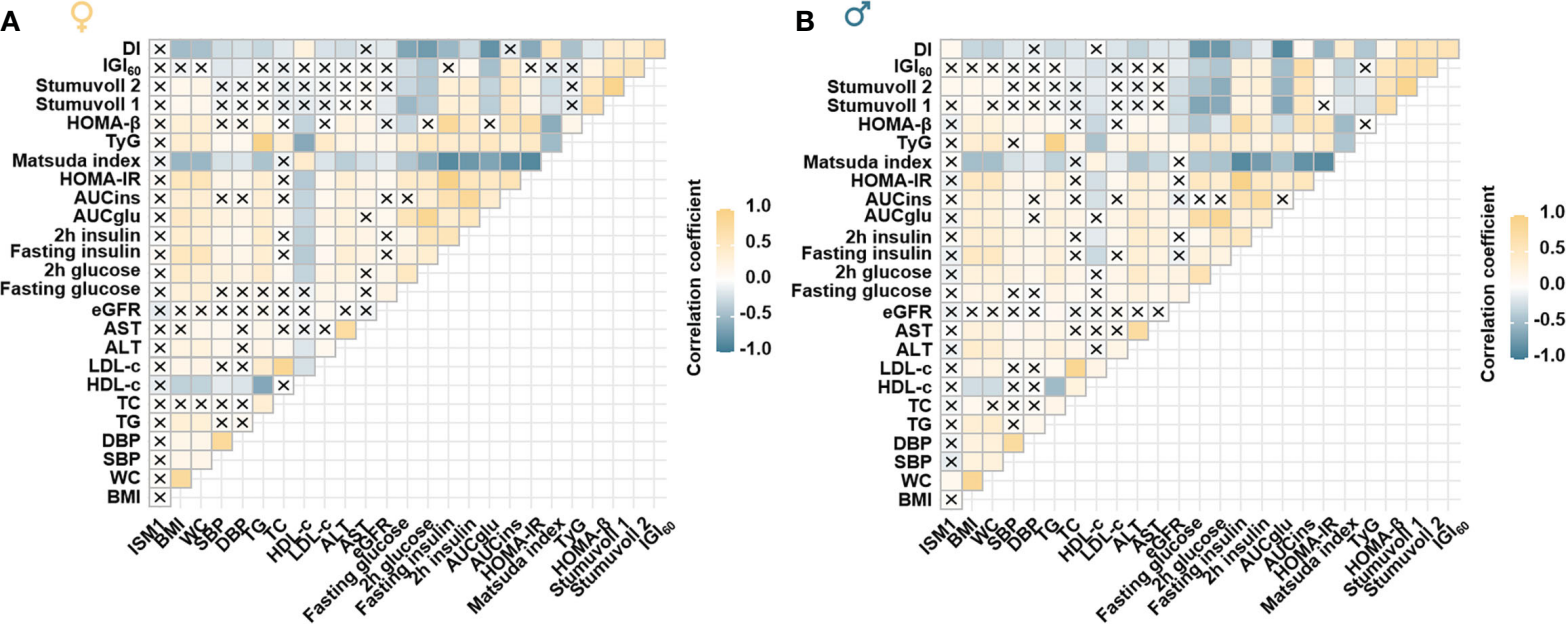
Association of ISM1 with metabolic parameters, insulin sensitivity and β-cell function. Heatmap showing the age-adjusted Spearman correlation coefficient between ISM1 and clinical parameters in **(A)** women and **(B)** men, respectively. Insulin sensitivity and β-cell function indices were based on the 2-h OGTT test. The symbol × indicates insignificant correlation. All *P* values were adjusted for age. WC, waist circumference.

Moreover, a distinct correlation between ISM1 and various metabolic parameters in men and women was found. Specifically, no significant correlation between ISM1 and any clinical features or metabolic indices related to glycemic control was found in women (all *P >*0.05, **Figure 4A**). On the contrary, ISM1 demonstrated a positive correlation with waist circumference and indices related to β-cell function, including Stumvoll 2 and disposition index (DI), in men after adjustment for age (*P <*0.05, **Figure 4B**).

**Figure 5:**
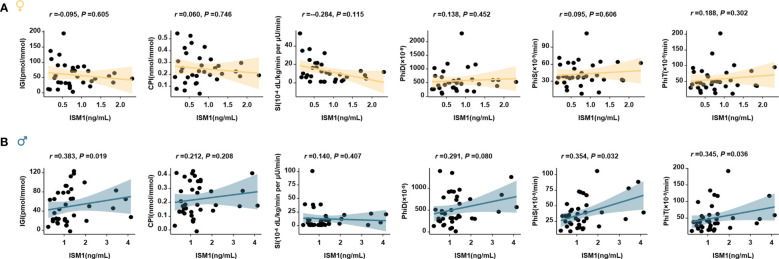
Association of ISM1 with oral minimal model-derived measurements. Linear regression models analyze the association of ISM1 with parameters related to insulin sensitivity and β-cell function in women **(A)** and men **(B)** with 3-h oral minimal model tests (38 men and 33 women). All *P* values were adjusted for age.

Notably, significant associations between ISM1 and β-cell function, in terms of IGI, PhiS and PhiT (oral minimal model-derived measurements), were also found in the subgroup of men who attended the 3 h-OGTT test (**Figure 5A**), whereas, no significant associations were observed between ISM1 and parameters related to insulin sensitivity in men and women (**Figure 5B**).

### Independent association of circulating ISM1 with IPH in men

**Figure 6:**
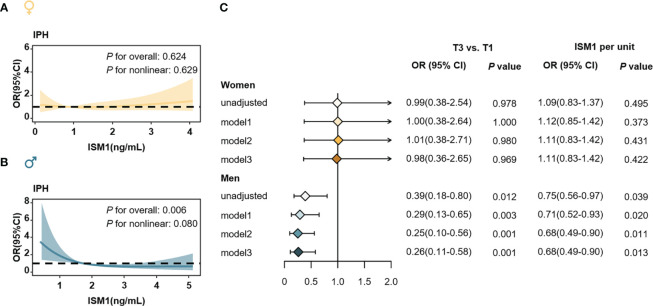
Isthmin-1 and Odds ratios of isolated post-challenge hyperglycemia. The association of serum ISM1 levels (as a continuous variable) with ORs of IPH in **(A)** women and **(B)** men, respectively. ORs (solid yellow and blue lines) and 95% CIs (light yellow and light blue areas) were derived from multivariable logistic regression using cubic natural spline. The *P* values were adjusted for age, history of hypertension, waist circumference, TG, HDL-c, eGFR and HOMA-IR. **(C)** Association of ISM1 (as a categorical variable) and the ORs of IPH in women (*up*) men (*down*). Model 1: adjusted for age, history of hypertension, TG, HDL-c and eGFR. Model 2: Model 1 plus waist circumference. Model 3: Model 2 plus HOMA-IR. IPH, isolated post-challenge hyperglycemia.

We then employed logistic regression models with cubic natural spline analyses to explore the association of serum ISM1 levels with ORs of IPH in men and women separately. In women, no association of ISM1 with the risk of IPH was found (**Figures 6A**, **C**), regardless of the menopause status (**Supplementary Figure S1**). However, in men, an increasing level of ISM1 was found to be significantly associated with a reduced risk of IPH (*P*=0.006, **Figure 6B**) after correcting for age, history of hypertension, waist circumference, TG, HDL-c, eGFR and HOMA-IR. Furthermore, when categorized into three groups according to the tertiles of ISM1 in our cohort, per unit increment in ISM1 was associated with a 32% (OR=0.68, 95% CI: 0.49-0.90) reduced risk of IPH in men. Compared to males in the lowest tertile of ISM1, those in the highest tertile of ISM1 experienced a greater than 70% (OR=0.26, 95% CI: 0.11-0.58) reduction in the risk of IPH after extensive adjustment for potential confounders (both *P*<0.05, **Figure 6C**).

Of note, the independent association of ISM1 with IPH in men remained robust in several sensitivity analyses, for instance, restricting the analysis to subjects with complete data of both 2 h-OGTT and HbA1c (n=428, 211 women and 217 men, **Supplementary Figure S2**) or expanding the analysis to the samples with ISM1 outliers (n=535, **Supplementary Figure S3**). Moreover, when using a broader definition of 2 h post-challenge hyperglycemia without considering the level of fasting glucose, the inverse association of ISM1 and risk for PH remained significant only in men (n=522, **Supplementary Figure S4**). Additionally, considering that most observed relationships between lifestyle and genetic factors and type 2 diabetes rarely exceeded 6.87 (the E-value for the adjusted OR of IPH with the highest tertile of ISM1) (9, 33), the E-value analysis suggested that no residual confounding factors remained in our study (**Supplementary Table S3**). Taken together, the above findings suggest that circulating ISM1 serves as an independent biomarker for IPH in men.

### Incremental discriminative value of ISM1 for IPH in men

To further examine the clinical relevance of ISM1, we then evaluated the additive value of ISM1 for the identification of diabetes and IPH on top of NCDRS, a widely used tool for the screening of undiagnosed diabetes in Chinese individuals (29, 34). Incorporation of ISM1 led to a 6% increase in the global performance for IPH differentiation, a modest and only marginally significant improvement (*P*=0.056, **Table 2**). Regarding the reclassification potential, which conveys more clinically important information (35), additional inclusion of ISM1 to the NCDRS resulted in 14% and 44% of the population being correctly reclassified as cases and non-cases, respectively, thus yielding an NRI of 58% (95% CI: 27%-89%, *P*<0.001) and IDI of 6.4% (95% CI: 2.7%-10.2%, *P*=0.001) for the total population (**Table 2**). Of note, compared to traditional clinical indices, such as fasting glucose and HbA1c, the incorporation of ISM1 into the NCDRS demonstrated a significantly better discrimination ability for IPH (**Table 2**). Moreover, such a substantial improvement in risk discrimination and reclassification was not materially changed in all sensitivity analyses, as evidenced by an increase in NRI from 36% to 65% and IDI ranging from 2.7% to 6.2% (all *P*<0.05,**Supplementary Tables S4–S6**). All models had acceptable calibration (Hosmer-Lemeshow statistic χ^2^ range from 4.21 to 13.36, all *P >*0.10).

**Table 2:** Additive differentiation and reclassification value of ISM1 for isolated post-challenge hyperglycemia on top of classical model.

Models	AUC (95% CI)	*P* value	Continuous net reclassification improvement (NRI)						Integrated discrimination improvement (95% CI), %	*P* value
			Cases, %		Non-cases, %		Full population (95% CI), %	*P* value		
			Up	Down	Up	Down				
NCDRS	0.73 (0.65-0.81)	Ref	Ref	Ref	Ref	Ref	Ref	Ref	Ref	Ref
Added variables										
FBG	0.73 (0.66-0.81)	0.120	57	43	60	40	34 (-30 to 74)	0.227	0.0 (-0.3 to 0.2)	0.759
HbA1c	0.72 (0.63-0.80)	0.374	59	41	61	39	39 (-20 to 89)	0.150	0.5 (-0.4 to 1.4)	0.245
ISM1	0.79 (0.72-0.85)	0.056	57	43	72	28	58 (27 to 89)	<0.001	6.4 (2.7 to 10.2)	0.001
FBG + HbA1c	0.72 (0.63-0.81)	0.447	51	49	55	45	12 (-9 to 82)	0.627	1.3 (-0.1 to 2.7)	0.076
FBG + ISM1	0.79 (0.73-0.86)	0.046	57	43	72	28	58 (27 to 92)	<0.001	6.4 (2.7 to 10.1)	0.001
FBG + HbA1c + ISM1	0.77 (0.70-0.84)	0.070	71	29	61	39	64 (26 to 100)	<0.001	6.0 (2.6 to 9.5)	0.001

NCDRS, New Chinese Diabetes Risk Score.

## Conclusions

In this deeply phenotyped cohort of medication-naïve community residents, we demonstrated an obvious sexual dimorphism in the distribution of circulating ISM1 and its association with β-cell function. Elevated ISM1 was independently associated with a decreased risk of IPH exclusively in men, but not in women. Previous studies have reported that ISM1 levels improved glucose tolerance and were associated with a lower risk of diabetes (11, 12). Conversely, other investigations found that serum ISM1 was significantly increased in patients with T2DM and correlated with the severity of albuminuria (36, 37). Such an ambiguous correlation might be mainly due to a limited sample size, methodology, and population characteristics, especially an incomplete adjustment for potential confounders. By selecting subjects without diagnosed diabetes and medication-naive subjects, extensively adjusting for potential confounders, and stratifying potential risk factors that may affect the ISM1-diabetes relationship, such as sex, we demonstrated a sexual difference of ISM1 with a decreased risk of IPH. Moreover, the incorporation of ISM1 into classical models for IPH screening yielded a significant improvement in the reclassification of Chinese subjects with IPH.

As an important subtype of diabetes, individuals with IPH have been reported to be at an increased risk for both incident cardiovascular disease and all-cause mortality compared to those with normal glucose levels (38, 39). Moreover, they also have an equal risk as those who have previously been diagnosed with diabetes mellitus (40). Additionally, incident diabetes and prediabetes in the elderly always manifest as post-challenge rather than fasting hyperglycemia (8). In our study, IPH accounted for up to 61.3% of newly diagnosed diabetes cases. Concordant with this result, the high prevalence of IPH of approximately 50-60% in subjects with newly diagnosed diabetes has also been described in previous population studies in Europe, Asia and China (7, 8, 41). Despite such a high burden, 2 h-OGTT remains an indispensable tool for the identification of IPH in current clinical practice, leaving an unmet need for novel biomarkers of IPH. In this regard, our findings that ISM1 is independently associated with β-cell function and serves as a sensitive biomarker for IPH in men are of major clinical importance. After adjustment for age, history of hypertension, waist circumference, TG, HDL-c, eGFR, and HOMA-IR, decreased ISM1 remained an independent risk factor for IPH in men. Notably, such an inverse association between circulating ISM1 and increased 2-h glucose persisted regardless of the fasting glucose level. Moreover, incorporation of ISM1 into the classical model for diabetes screening led to a net reclassification of 58% for the total population in our study, which means that approximately three in five subjects with undiagnosed diabetes could be identified by additional inclusion of ISM1, and appropriate interventions can thus be implemented in time to avoid severe and potentially irreversible complications of their unmanaged hyperglycemia.

As a newly identified adipokine, the circulating level of ISM1 and its distribution characteristics have been poorly investigated. Overall, the median level of ISM1 was 1.3 ng/L in our study, slightly lower than the level of 3.72 ng/mL in subjects with a significantly higher BMI (27 in the previous study *vs.* 25 in our cohort) (12), but comparable to the level of approximately 0.9-1.1 ng/mL found in individuals with diabetes and a similar BMI range to ours (36). Although it was reported to be positively associated with BMI in females (11), no significant associations with BMI were found in our study. To our knowledge, we are the first to report a sexual difference in the distribution of ISM1. In extensively adjusted models, we observed a significant difference in ISM1, with higher levels in men. Sexual differences have also been reported for several adipokines, such as adiponectin, leptin, and vaspin levels (42–44). Our finding of sexual dimorphism of ISM1 was further supported by a recent report that ISM1 was only significantly higher in pubertal boys with obesity (45). Such a sex difference in the distribution of ISM1 and other adipokines may partially account for the difference in insulin sensitivity and β-cell function observed between men and women.

Mechanistically, IPH was reported primarily due to deterioration of insulin secretion and, to a lesser extent, to insulin resistance (46, 47). Consistently, no obvious association between circulating ISM1 and insulin sensitivity indices, either based on the basal model or derived from glucose stimulation, was found in our study or the previous study (11, 12, 37, 45). In contrast, a significant positive association of ISM1 levels and β-cell function was found in men by several methodologies, including the Stumvoll indices and oral minimal model test-derived indices, which quantitatively simulate the complex process of glucose metabolism with a mathematical model and analog β-cell responsibility (20, 21). These data were well in line with the previous animal study revealed that ISM1 served as a secreted factor that exogenously and endogenously controls glucose uptake *in vitro*, which could activate the PI3K-AKT signaling pathway independently of insulin, and insulin-like growth factor receptors improved glucose tolerance (11).Thus, the protective role of ISM1 and IPH might be related to insulin secretion ability. However, more in-depth studies considering the role of ISM1 in insulin secretion are needed in the future.

Our study has several strengths, including the employment of more accurate estimates of pancreatic β-cell function derived from both the 2 h-OGTT test and oral minimal model, such as Stumvoll first and second phase secretion index, PhiD, PhiS and PhiT, other than HOMA-β which only takes into account the basal glucose and insulin concentrations. Such indices based on mathematical models upon glucose stimulation are more sensitive for probing mild defects in β-cell function and provide a more accurate evaluation of the complex process of glucose metabolism (20, 21). Moreover, we also included various sensitivity analyses to ensure the robustness of the findings. Nevertheless, several limitations of this study should be acknowledged. First, our participants were only Chinese recruited from a single center, and thus, further validation in larger and more diverse ethnicities is warranted. Second, despite an extensive adjustment, residual confounding was still possible due to the nature of observational studies. However, the results remained basically the same in various sensitivity analyses. Moreover, considering the large E value that a potentially unmeasured confounding factor would need to deviate from our findings, the E value analysis suggested that no residual confounding factors remained in our study.

In conclusion, our study provides evidence that there is sexual dimorphism in the distribution of circulating ISM1. ISM1 independently associates with β-cell function in men and functions as an easy-to-use tool for the identification of male subjects with high risk of IPH in clinical practice.

## Data availability statement

The raw data supporting the conclusions of this article will be made available by the authors, without undue reservation.

## Ethics statement

This study complied with the Declaration of Helsinki and was approved by the School of Public Health Institutional Review Board of Sun Yat-sen University. All participants provided written informed consent before data collection. The studies were conducted in accordance with the local legislation and institutional requirements. The participants provided their written informed consent to participate in this study.

## Author contributions

JF: Conceptualization, Data curation, Formal analysis, Funding acquisition, Investigation, Methodology, Writing – original draft, Writing – review & editing. JHe: Data curation, Formal analysis, Investigation, Methodology, Writing – original draft, Writing – review & editing. JZ: Data curation, Investigation, Methodology, Writing – review & editing. JY: Data curation, Investigation, Writing – review & editing. JJ: Data curation, Investigation, Writing – review & editing. JHu: Data curation, Investigation, Writing – review & editing. ZH: Data curation, Investigation, Writing – review & editing. ZZ: Data curation, Investigation, Writing – review & editing. WL: Data curation, Investigation, Writing – review & editing. MX: Resources, Writing – review & editing. YL: Conceptualization, Funding acquisition, Project administration, Resources, Supervision, Writing – review & editing.
