# Supplementary material for: Sex-specific association of circulating Isthmin-1 with isolated post-challenge hyperglycemia
Source: Front Endocrinol (Lausanne). 2024 Jul 25;15:1394190. doi: 10.3389/fendo.2024.1394190 (PMC11306075; doi:10.3389/fendo.2024.1394190)
Supplement: Supplementary file 1 [file DataSheet_1.docx]

**SUPPLEMENTAL MATERIAL TO**

**Sex-specific association of circulating Isthmin-1 with isolated**

**post-challenge hyperglycemia**

***Abbreviated title:*** ISM1 and post-challenge hyperglycemia

Jiahua Fan^1,2,*^, Jialin He^1,2,*^, Jiangyuan Zhu^1,2^, Jialu Yang^1,2^, Jingmeng Ju^1,2^, Jingyi Huang^1,2^, Zhihao Huang^1^, Zhuoyu Zhang^1,2^, Wenkang Li^1^, Min Xia^1,2^, Yan Liu^1,2,#^

^1^Guangdong Provincial Key Laboratory of Food, Nutrition and Health, Guangzhou, P.R. China

^2^Department of Nutrition, School of Public Health, Sun Yat-sen University, Guangzhou, P.R. China

^*^J.F., J.H. contributed equally to this work.

Corresponding author: Dr. Yan Liu, [liuyan215@mail.sysu.edu.cn](mailto:liuyan215@mail.sysu.edu.cn), Department of Nutrition, School of Public Health, Sun Yat-sen University

**Supplementary Table S1 Participant characteristics stratified by ISM1 tertiles in women**

|  | **Total (n = 254)** | **T1 (n=84)** | **T2 (n=85)** | **T3 (n=85)** | ***P* value** |
| --- | --- | --- | --- | --- | --- |
| ISM1, ng/mL | 0.88 (0.22-1.72) | 0.16 (0.14-0.22) | 0.88 (0.59-1.10) | 2.22 (1.72-3.12) | < 0.001 |
| **Demographics and laboratory variables** | | | | | |
| Age, years | 47.63 ± 7.23 | 46.73 ± 6.56 | 48.09 ± 7.78 | 48.07 ± 7.29 | 0.373 |
| BMI, kg/m2 | 24.41 ± 3.33 | 24.6 ± 3.78 | 24.24 ± 3.21 | 24.38 ± 3.00 | 0.788 |
| Waist circumference, cm | 82.89 ± 8.81 | 82.50 ± 9.76 | 82.38 ± 8.05 | 83.80 ± 8.58 | 0.507 |
| SBP, mmHg | 120 (109-133) | 117 (109-126) | 124 (110-140) | 120 (109-135) | 0.080 |
| DBP, mmHg | 80 (75-87) | 79 (75-86) | 81 (73-90) | 80 (75-86) | 0.678 |
| TG, mmol/L | 1.28 (0.93-1.92) | 1.27 (0.84-1.84) | 1.21 (0.96-1.90) | 1.34 (0.93-1.98) | 0.697 |
| TC, mmol/L | 5.20 (4.72-6.03) | 5.20 (4.69-6.04) | 5.07 (4.72-5.97) | 5.33 (4.79-6.08) | 0.415 |
| HDL-c, mmol/L | 1.43 (1.21-1.66) | 1.48 (1.28-1.80) | 1.42 (1.21-1.61) | 1.37 (1.19-1.61) | 0.091 |
| LDL-c, mmol/L | 3.28 ± 0.83 | 3.32 ± 0.79 | 3.13 ± 0.78 | 3.39 ± 0.90 | 0.099 |
| eGFR, mL/min/1.72m2 | 99.29 (87.16-109.76) | 104.61 (91.23-111.57) | 96.28 (86.54-108.53) | 97.36 (84.29-106.06) | 0.016 |
| Fasting glucose, mmol/L | 5.40 (5.00-5.81) | 5.47 (5.19-5.78) | 5.40 (4.97-6.00) | 5.34 (5.00-5.73) | 0.713 |
| 2-h glucose, mmol/L | 7.80 (6.68-9.50) | 7.85 (6.31-9.35) | 8.17 (6.70-10.02) | 7.70 (6.90-9.10) | 0.588 |
| Fasting insulin, mU/L | 6.85 (4.92-10.7) | 6.33 (3.95-9.15) | 7.46 (5.35-12.6) | 6.92 (5.2-10.71) | 0.067 |
| 2-h insulin, mU/L | 52.62 (33.50-93.13) | 50.70 (34.90-89.59) | 59.71 (33.02-86.72) | 50.64 (33.61-93.99) | 0.920 |
| HbA1c, % ^#^ | 5.7 (5.4-6.0) | 5.6 (5.4-5.9) | 5.8 (5.6-6.2) | 5.8 (5.4-6.1) | 0.007 |
| HbA1c, mmol/mol ^#^ | 39 (36-42) | 38 (36-41) | 40 (38-44) | 40 (36-43) | 0.007 |
| AUCglu, mmol/L×h | 16.42 (13.76-19.89) | 16.21 (14.10-18.50) | 17.00 (13.06-21.06) | 16.15 (13.73-19.88) | 0.780 |
| AUC_ins_, mU/L×h | 98.68 (66.75-148.90) | 102.04 (66.26-140.47) | 90.13 (63.79-162.96) | 98.49 (70.27-139.50) | 0.953 |
| **Insulin sensitivity** |  |  |  |  |  |
| HOMA-IR | 1.65 (1.14-2.76) | 1.53 (0.96-2.48) | 1.80 (1.25-3.05) | 1.65 (1.25-2.71) | 0.120 |
| TyG index | 3.76 ± 0.26 | 3.75 ± 0.27 | 3.77 ± 0.27 | 3.77 ± 0.24 | 0.807 |
| Matsuda index | 4.44 (2.79-6.77) | 4.69 (3.01-7.11) | 4.17 (2.46-7.38) | 4.60 (2.91-6.27) | 0.449 |
| **β-cell function** |  |  |  |  |  |
| HOMA-β | 68.86 (48.66-116.51) | 61.89 (44.45-94.99) | 78.39 (50.47-128.61) | 71.21 (49.94-129.84) | 0.027 |
| Stumvoll 1 | 759.77 (497.1-987.82) | 767.24 (537.47-906.28) | 776.09 (479.91-1008.17) | 735.3 (496.92-1045.28) | 0.923 |
| Stumvoll 2 | 217.14 (154.02-268.63) | 221.99 (165.24-256.07) | 215.23 (145.78-267.6) | 214.94 (163.06-278.13) | 0.910 |
| IGI60 | 14.12 (8.25-25.03) | 14.21 (10.04-23.04) | 13.71 (7.3-25.24) | 14.69 (8.09-26.2) | 0.850 |
| DI | 27.56 (19.78-35.87) | 29.07 (21.35-38.29) | 26.87 (17.24-35.84) | 27.83 (21.75-34.86) | 0.433 |
| **Lifestyle and Comorbidities, n (%)** | | | | | |
| Current smoker | 1 (0.4) | 0 (0) | 1 (1.2) | 0 (0) | 1.000 |
| Current drinker | 3 (1.2) | 1 (1.2) | 1 (1.2) | 1 (1.2) | 1.000 |
| Physical activity | 88 (34.6) | 40 (47.1) | 23 (27.1) | 25 (29.1) | 0.010 |
| Menopausal | 96 (37.8) | 26 (31.0) | 35 (41.2) | 35 (41.2) | 0.287 |
| Hypertension | 75 (29.5) | 20 (23.8) | 31 (36.5) | 24 (28.2) | 0.187 |
| Dyslipidemia | 95 (37.4) | 31 (36.9) | 28 (32.9) | 36 (42.4) | 0.445 |
| Prediabetes | 113 (44.5) | 36 (49.2) | 38 (44.7) | 39 (45.9) | 0.924 |
| Impaired glucose tolerance | 82 (32.3) | 27 (32.1) | 30 (35.3) | 25 (29.4) | 0.714 |
| Isolated impaired glucose tolerance | 52 (20.5) | 15 (17.9) | 20 (23.5) | 17 (20.0) | 0.653 |
| Diabetes | 49 (19.3) | 17 (20.2) | 16 (18.8) | 16 (18.8) | 0.965 |
| Post-challenge hyperglycemia | 47 (18.5) | 16 (19.0) | 16 (18.8) | 15 (17.6) | 0.969 |
| Isolated post-challenge hyperglycemia | 29 (11.4) | 10 (11.9) | 9 (10.6) | 10 (11.8) | 0.957 |

Data were shown as mean±SD, or median (lower quartile, upper quartile), or n and percent (%). *P* value indicated the difference between subjects with different tertiles of ISM1. ^#^The total number was 211.

**Supplementary Table S2 Participant characteristics stratified by ISM1 tertiles in men**

|  | **Total (n = 268)** | **T1 (n=89)** | **T2 (n=89)** | **T3 (n=90)** | ***P* value** |
| --- | --- | --- | --- | --- | --- |
| ISM1, ng/mL | 1.74 (1.02-2.63) | 0.78 (0.62-1.01) | 1.74 (1.42-2.04) | 3.30 (2.63-4.06) | < 0.001 |
| **Demographics and laboratory variables** | | | | | |
| Age, years | 46.62 ± 7.20 | 45.83 ± 7.49 | 46.18 ± 7.44 | 47.83 ± 6.56 | 0.138 |
| BMI, kg/m^2^ | 25.92 ± 3.14 | 25.75 ± 3.11 | 26.01 ± 3.25 | 25.99 ± 3.08 | 0.830 |
| Waist circumference, cm | 91.45 ± 8.34 | 90.32 ± 9.20 | 91.27 ± 7.97 | 92.75 ± 7.67 | 0.144 |
| SBP, mmHg | 127 (118-138) | 130 (121-139) | 126 (115-137) | 124 (115-136) | 0.029 |
| DBP, mmHg | 86 (81-92) | 87 (82-95) | 86 (80-93) | 86 (78-91) | 0.149 |
| TG, mmol/L | 2.01 (1.33-2.85) | 2.07 (1.31-2.88) | 1.92 (1.33-2.66) | 2.08 (1.38-3.13) | 0.848 |
| TC, mmol/L | 5.09 (4.54-5.89) | 5.24 (4.55-5.93) | 4.97 (4.55-5.56) | 5.11 (4.50-5.98) | 0.513 |
| HDL-c, mmol/L | 1.12 (0.99-1.28) | 1.12 (0.99-1.30) | 1.11 (1.01-1.27) | 1.12 (0.99-1.30) | 0.817 |
| LDL-c, mmol/L | 3.32 ± 0.78 | 3.34 ± 0.83 | 3.31 ± 0.79 | 3.32 ± 0.73 | 0.958 |
| eGFR, mL/min/1.72m^2^ | 93.45 (77.7-105.29) | 96.36 (81.42-109.04) | 88.13 (75.10-105.14) | 90.17 (74.39-102.52) | 0.059 |
| Fasting glucose, mmol/L | 5.52 (5.08-6.10) | 5.66 (5.10-6.00) | 5.50 (4.97-6.16) | 5.50 (5.13-6.10) | 0.705 |
| 2-h glucose, mmol/L | 8.50 (6.70-11.56) | 9.67 (6.78-12.80) | 8.00 (6.68-10.40) | 8.40 (6.55-10.57) | 0.087 |
| Fasting insulin, mU/L | 8.08 (5.67-11.95) | 8.42 (5.87-12.32) | 8.73 (6.2-12.48) | 7.23 (5.31-10.34) | 0.094 |
| 2-h insulin, mU/L | 57.11 (31.43-94.18) | 51.21 (31.64-91.95) | 60.43 (32.43-94.88) | 55.05 (30.46-97.72) | 0.819 |
| HbA1c, % ^#^ | 5.8 (5.6-6.2) | 5.8 (5.6-6.2) | 5.8 (5.6-6.2) | 5.8 (5.4-6.1) | 0.676 |
| HbA1c, mmol/mol ^#^ | 40 (38-44) | 40 (38-44) | 40 (38-44) | 40 (36-43) | 0.676 |
| AUC_glu_, mmol/L×h | 18.18 (15.58-21.96) | 19.01 (16.50-23.30) | 17.38 (15.26-21.64) | 17.96 (15.26-20.91) | 0.202 |
| AUC_ins_, mU/L×h | 106.44 (69.89-172.39) | 101.26 (60.02-170.94) | 109.65 (71.62-174.75) | 107.64 (73.54-152.83) | 0.576 |
| **Insulin sensitivity** |  |  |  |  |  |
| HOMA-IR | 2.00 (1.39-3.07) | 2.12 (1.44-3.17) | 2.06 (1.47-3.43) | 1.89 (1.31-2.55) | 0.171 |
| TyG index | 3.95 ± 0.29 | 3.95 ± 0.26 | 3.95 ± 0.28 | 3.96 ± 0.32 | 0.945 |
| Matsuda index | 3.77 (2.50-5.33) | 3.78 (2.43-5.43) | 3.27 (2.35-5.27) | 3.98 (2.80-5.30) | 0.432 |
| **β-cell function** |  |  |  |  |  |
| HOMA-β | 83.94 (53.2-124.95) | 85.43 (51.15-124.88) | 90.22 (63.97-135.77) | 74.46 (49.15-108.71) | 0.058 |
| Stumvoll 1 | 738.41 (281.17-1081.03) | 652.01 (177.85-952.77) | 834.63 (397.8-1154.37) | 745.7 (331.12-1120.22) | 0.045 |
| Stumvoll 2 | 214.72 (140.29-286.11) | 184.51 (63.2-265.02) | 230.79 (165.24-298.7) | 225.65 (142.15-286.78) | 0.018 |
| IGI60 | 12.24 (6.63-19.57) | 11.41 (5.76-17.03) | 12.86 (6.80-20.04) | 12.97 (7.28-21.72) | 0.466 |
| DI | 23.24 (15.27-31.32) | 21.96 (13.96-26.94) | 23.85 (15.76-33.97) | 25.19 (15.97-34.39) | 0.085 |
| **Lifestyle and Comorbidities, n (%)** | | | | | |
| Current smoker | 94 (35.1) | 30 (33.7) | 39 (43.8) | 25 (27.8) | 0.076 |
| Current drinker | 57 (21.3) | 19 (21.3) | 20 (22.5) | 18 (20) | 0.921 |
| Physical activity | 89 (33.2) | 27 (30.3) | 30 (33.3) | 32 (35.6) | 0.758 |
| Hypertension | 129 (48.1) | 44 (49.4) | 39 (43.8) | 46 (51.1) | 0.593 |
| Dyslipidemia | 168 (62.7) | 62 (69.7) | 53 (59.6) | 53 (58.9) | 0.249 |
| Prediabetes | 107 (39.9) | 34 (38.2) | 34 (38.2) | 39 (43.3) | 0.720 |
| Impaired glucose tolerance | 84 (31.3) | 25 (28.1) | 28 (31.5) | 31 (34.4) | 0.657 |
| Isolated impaired glucose tolerance | 42 (21.8) | 14 (25.5) | 12 (17.4) | 16 (23.2) | 0.523 |
| Diabetes | 75 (28.0) | 34 (38.2) | 20 (22.5) | 21 (23.3) | 0.031 |
| Post-challenge hyperglycemia | 73 (27.2) | 34 (38.2) | 19 (21.3) | 20 (22.2) | 0.017 |
| Isolated post-challenge hyperglycemia | 47 (17.5) | 27 (30.3) | 7 (7.9) | 13 (14.4) | < 0.001 |

Data were shown as mean±SD, or median (lower quartile, upper quartile), or n and percent (%). *P* value indicated the difference between subjects with different tertiles of ISM1. ^#^The total number was 217.

**Supplementary Table S3 E-values to assess minimum multivariable adjusted OR that an unmeasured confounder would need to have with both the exposure and outcome to fully explain the observed relationships between ISM1 and IPH**

| **ISM1 levels** | **Adjusted OR** | **Upper 95%CI** | **E-Value for adjusted OR** | **E-Value for upper CI** |
| --- | --- | --- | --- | --- |
| T1 | 1.00 | - | Ref | - |
| T2 | 0.16 | 0.41 | 11.98 | 4.31 |
| T3 | 0.27 | 0.61 | 6.87 | 2.66 |
| Per unit | 0.69 | 0.91 | 2.26 | 1.43 |

**Supplementary Table S4 Additive differentiation and reclassification value of ISM1 for isolated post-challenge hyperglycemia on top of classical model in men with complete data of HbA1c**

| **Models** | **AUC**  **(95% CI)** | ***P* value** | **Continuous Net Reclassification improvement (NRI)** | | | | | | **Integrated discrimination improvement (95% CI), %** | ***P* value** |
| --- | --- | --- | --- | --- | --- | --- | --- | --- | --- | --- |
|  |  |  | **Cases, %** | | **Non-cases, %** | | **Full population**  **(95% CI), %** | ***P* value** |  |  |
|  |  |  | **Up** | **Down** | **Up** | **Down** |  |  |  |  |
| NCDRS | 0.64  (0.55-0.74) | Ref | Ref | Ref | Ref | Ref | Ref | Ref | Ref | Ref |
| *Added variables* |  |  |  |  |  |  |  |  |  |  |
| FBG | 0.65  (0.56-0.74) | 0.264 | 54 | 46 | 60 | 40 | 27 (-30 to 69) | 0.327 | 0.0 (-0.2 to 0.2) | 0.781 |
| HbA1c | 0.65  (0.55-0.74) | 0.796 | 45 | 54 | 47 | 53 | -14 (-36 to 50) | 0.532 | 0.2 (-0.2 to 0.6) | 0.342 |
| ISM1 | 0.74  (0.65-0.83) | 0.078 | 64 | 36 | 68 | 32 | 65 (29 to 101) | <0.001 | 5.8 (2.4 to 9.3) | 0.001 |
| FBG + HbA1c | 0.66  (0.57-0.75) | 0.596 | 57 | 43 | 57 | 43 | 28 (-22 to 75) | 0.228 | 0.5 (-0.6 to 1.5) | 0.357 |
| FBG + ISM1 | 0.74  (0.65-0.83) | 0.073 | 64 | 36 | 68 | 32 | 65 (25 to 104) | <0.001 | 5.9 (2.4 to 9.3) | 0.001 |
| FBG + HbA1c + ISM1 | 0.75  (0.65-0.85) | 0.082 | 68 | 32 | 67 | 33 | 70 (30 to 112) | <0.001 | 7.5 (3.5 to 11.5) | <0.001 |

NCDRS**:** New Chinese Diabetes Risk Score.

**Supplementary Table S5 Additive differentiation and reclassification value of ISM1 for isolated post-challenge hyperglycemia on top of classical model in men with ISM1 outliers**

| **Models** | **AUC**  **(95% CI)** | ***P* value** | **Continuous Net Reclassification improvement (NRI)** | | | | | | **Integrated discrimination improvement (95% CI), %** | ***P* value** |
| --- | --- | --- | --- | --- | --- | --- | --- | --- | --- | --- |
|  |  |  | **Cases, %** | | **Non-cases, %** | | **Full population**  **(95% CI), %** | ***P* value** |  |  |
|  |  |  | **Up** | **Down** | **Up** | **Down** |  |  |  |  |
| NCDRS | 0.72  (0.65-0.80) | Ref | Ref | Ref | Ref | Ref | Ref | Ref | Ref | Ref |
| *Added variables* |  |  |  |  |  |  |  |  |  |  |
| FBG | 0.73  (0.65-0.80) | 0.213 | 57 | 43 | 61 | 39 | 36 (-32 to 73) | 0.203 | 0.0 (-0.4 to 0.3) | 0.912 |
| HbA1c | 0.72  (0.63-0.80) | 0.240 | 61 | 39 | 62 | 38 | 46 (-18 to 89) | 0.069 | 0.7 (-0.3 to 1.6) | 0.160 |
| ISM1 | 0.78  (0.71-0.85) | 0.054 | 57 | 43 | 72 | 28 | 58 (26 to 89) | <0.001 | 6.2 (2.5 to 9.9) | 0.001 |
| FBG + HbA1c | 0.72  (0.63-0.80) | 0.391 | 51 | 49 | 54 | 46 | 11 (-9 to 83) | 0.634 | 1.4 (0.0 to 2.9) | 0.057 |
| FBG + ISM1 | 0.78  (0.72-0.85) | 0.042 | 57 | 43 | 72 | 28 | 58 (26 to 92) | <0.001 | 6.2 (2.5 to 9.8) | 0.001 |
| FBG + HbA1c + ISM1 | 0.76  (0.68-0.83) | 0.086 | 61 | 39 | 65 | 35 | 52 (27 to 97) | 0.004 | 5.3 (2.0 to 8.7) | 0.002 |

NCDRS**:** New Chinese Diabetes Risk Score.

**Supplementary Table S6 Additive differentiation and reclassification value of ISM1 for post-challenge hyperglycemia on top of classical model**

| **Models** | **AUC**  **(95% CI)** | ***P* value** | **Continuous Net Reclassification improvement (NRI)** | | | | | | **Integrated discrimination improvement (95% CI), %** | ***P* value** |
| --- | --- | --- | --- | --- | --- | --- | --- | --- | --- | --- |
|  |  |  | **Cases, %** | | **Non-cases, %** | | **Full population**  **(95% CI), %** | ***P* value** |  |  |
|  |  |  | **Up** | **Down** | **Up** | **Down** |  |  |  |  |
| NCDRS | 0.72  (0.65-0.79) | Ref | Ref | Ref | Ref | Ref | Ref | Ref | Ref | Ref |
| *Added variables* |  |  |  |  |  |  |  |  |  |  |
| FBG | 0.87  (0.83-0.92) | <0.001 | 73 | 27 | 78 | 22 | 102 (76 to 125) | <0.001 | 27.0 (19.8 to 34.2) | <0.001 |
| HbA1c | 0.86  (0.80-0.92) | 0.002 | 75 | 25 | 81 | 19 | 112 (78 to 137) | <0.001 | 28.5 (21.3 to 35.8) | <0.001 |
| ISM1 | 0.73  (0.67-0.80) | 0.347 | 46 | 54 | 72 | 28 | 36 (11 to 65) | 0.001 | 2.7 (0.4 to 4.9) | 0.023 |
| FBG + HbA1c | 0.89  (0.85-0.94) | <0.001 | 75 | 25 | 81 | 19 | 112 (89 to 144) | <0.001 | 32.9 (25.1 to 40.7) | <0.001 |
| FBG + ISM1 | 0.90  (0.86-0.94) | <0.001 | 74 | 26 | 78 | 22 | 104 (83 to 134) | <0.001 | 32.3 (25.1 to 39.5) | <0.001 |
| FBG + HbA1c + ISM1 | 0.91  (0.87-0.95) | <0.001 | 81 | 19 | 80 | 20 | 122 (93 to 148) | <0.001 | 38.1 (30.5 to 45.8) | <0.001 |

NCDRS**:** New Chinese Diabetes Risk Score.


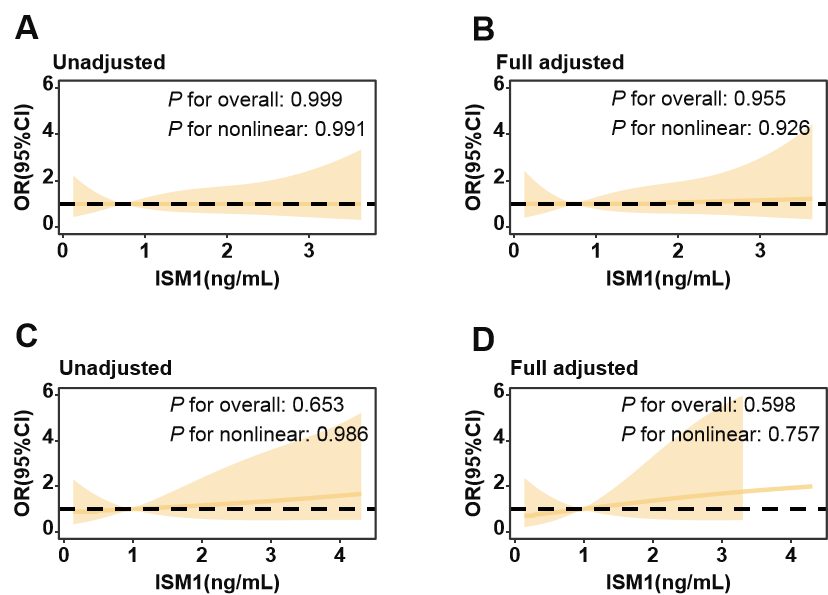


**Supplementary Fig. 1 Association of ISM1 with IPH in women subgroup by menopausal status.** The association of serum ISM1 levels (as a continuous variable) with ORs of IPH in (A and B) women without post-menopausal and (C and D) with post-menopausal, respectively. ORs (solid yellow lines) and 95% CIs (light yellow areas) were derived from multivariable logistic regression using cubic natural spline. A and C unadjusted. B and D adjusted for age, history of hypertension, TG, HDL-c, eGFR, waist circumference and HOMA-IR. IPH, isolated post-challenge hyperglycemia.


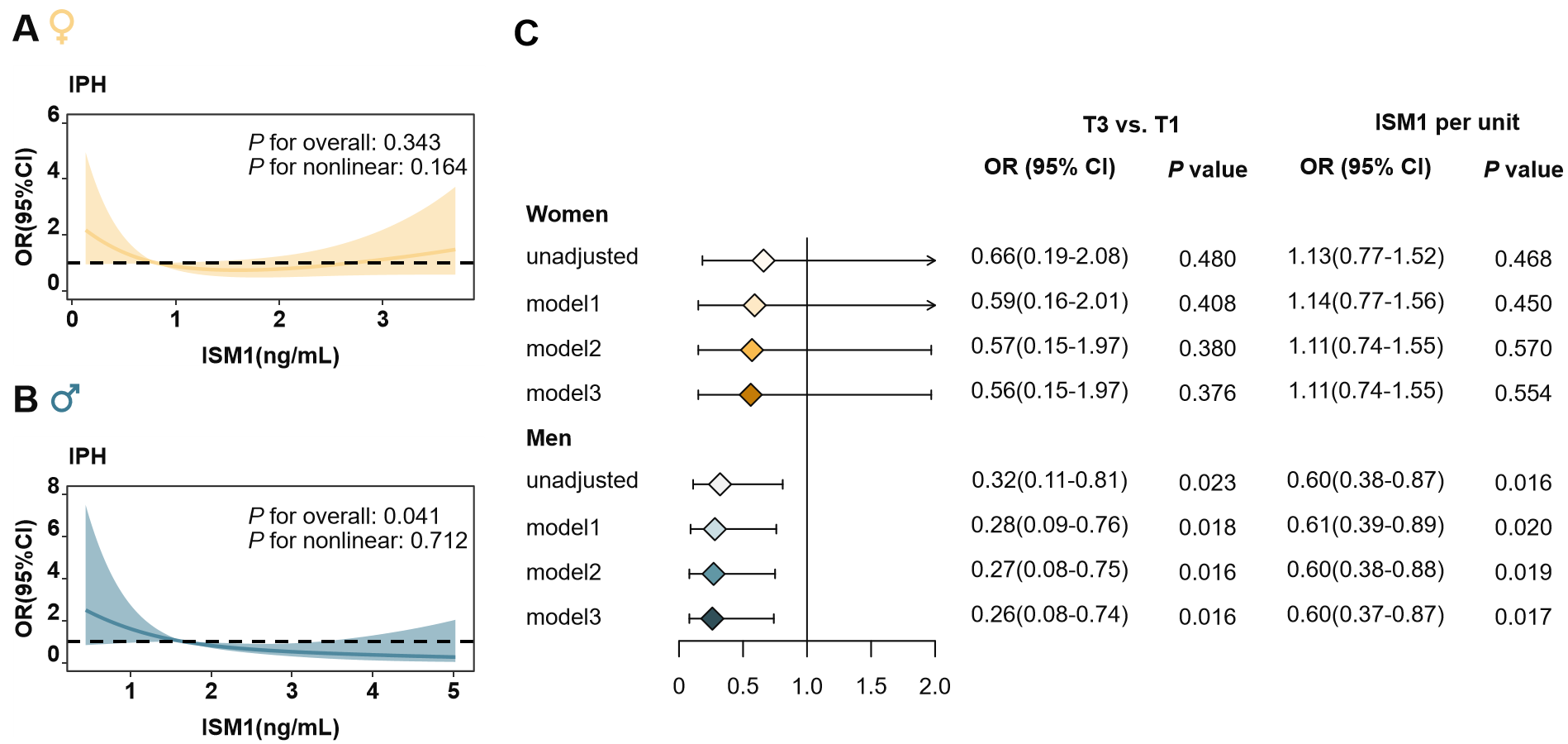


**Supplementary Fig. 2 Association of ISM1 with IPH in men and women: sensitivity analysis 1.** Only subjects with complete data of 2h-OGTT and HbA1c were included in this analysis (*n*=428). The association of serum ISM1 levels (as a continuous variable) with ORs of IPH in (A) women and (B) men, respectively. ORs (solid yellow and blue lines) and 95% CIs (light yellow and light blue areas) were derived from multivariable logistic regression using cubic natural spline. (C) Association of ISM1 (as a categorical variable) and the ORs of IPH in women (*upper*) and men (*lower*). Model 1: adjusted for age, history of hypertension, TG, HDL-c and eGFR. Model 2: Model 1 plus waist circumference. Model 3: Model 2 plus HOMA-IR. IPH, isolated post-challenge hyperglycemia.


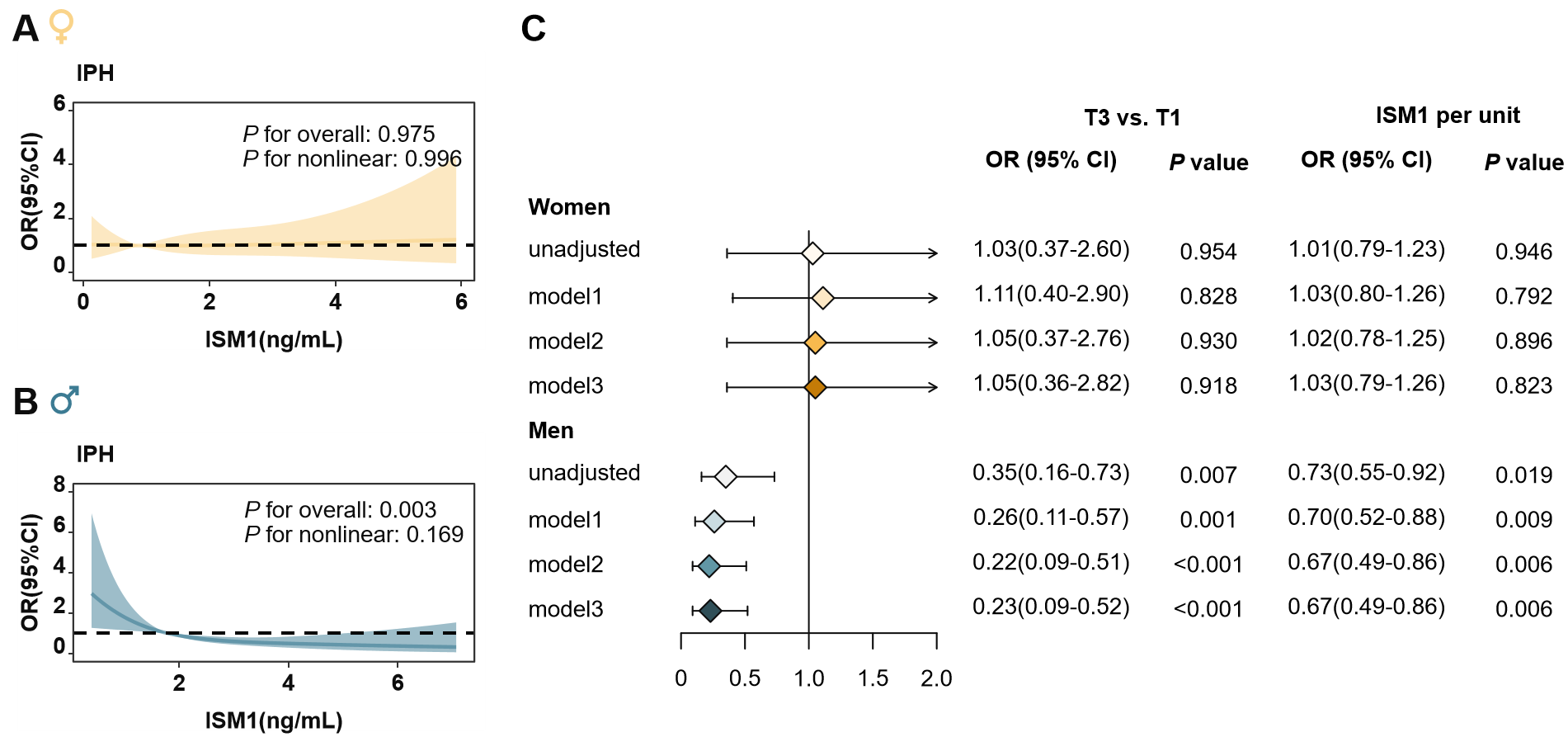


**Supplementary Fig. 3 Association of ISM1 with IPH in men and women: sensitivity analysis 2.** Subjects with outliers of ISM1 levels were included in this analysis (*n*=535). The association of serum ISM1 levels (as a continuous variable) with ORs of IPH in (A) women and (B) men, respectively. ORs (solid yellow and blue lines) and 95% CIs (light yellow and light blue areas) were derived from multivariable logistic regression using cubic natural spline. (C) Association of ISM1 (as a categorical variable) and the ORs of IPH in women (*upper*) and men (*lower*). Model 1: adjusted age, history of hypertension, TG, HDL-c and eGFR. Model 2: Model 1 plus waist circumference. Model 3: Model 2 plus HOMA-IR. IPH, isolated post-challenge hyperglycemia.

**
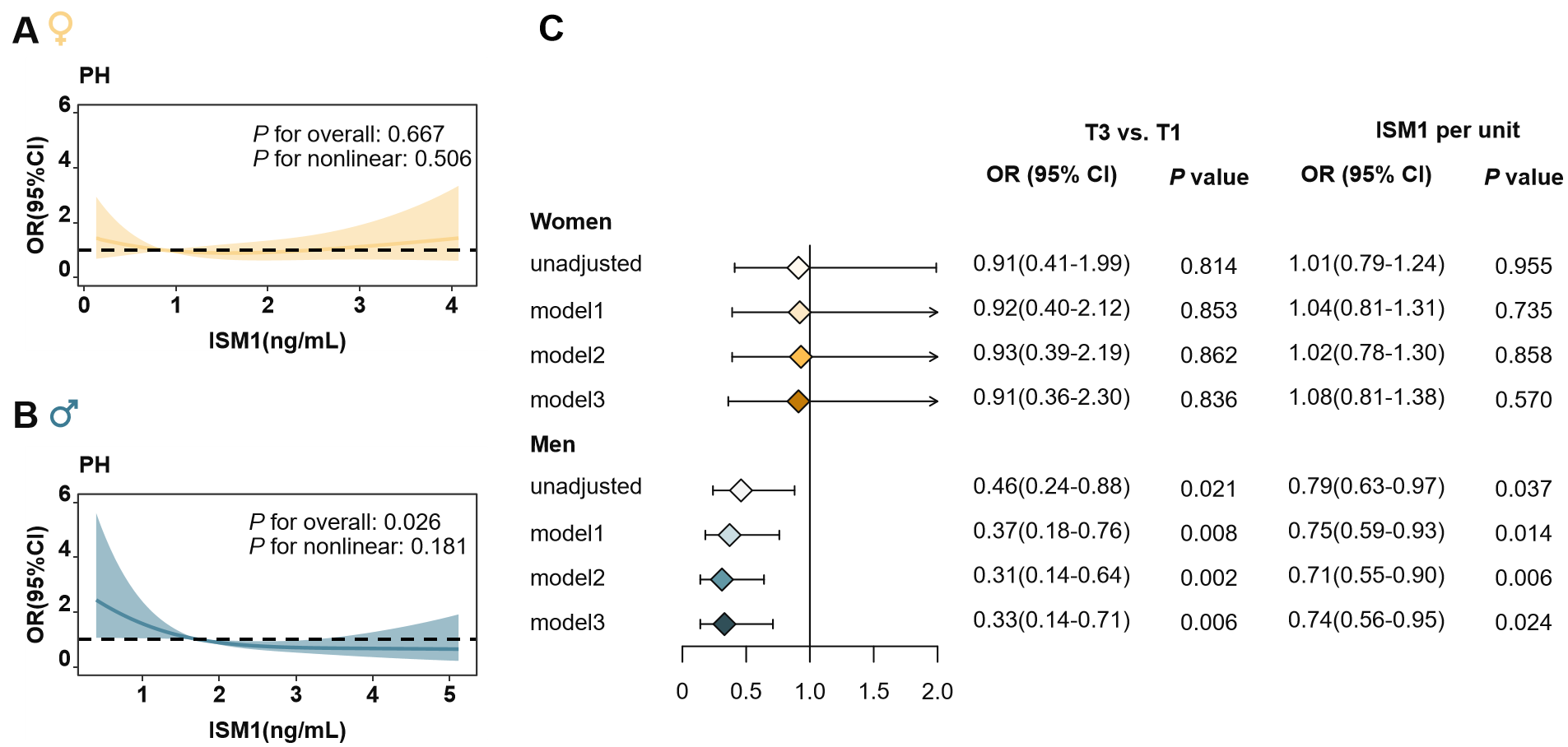
**

**Supplementary Fig. 4 Association of ISM1 with PH in men and women: sensitivity analysis 3.** A total of 522 subjects were included in this analysis. The association of serum ISM1 levels (as a continuous variable) with ORs of PH in (A) women and (B) men, respectively. ORs (solid yellow and blue lines) and 95% CIs (light yellow and light blue areas) were derived from multivariable logistic regression using cubic natural spline. (C) Association of ISM1 (as a categorical variable) and the ORs of PH in women (*upper*) and men (*lower*). Model 1: adjusted age, history of hypertension, TG, HDL-c and eGFR. Model 2: Model 1 plus waist circumference. Model 3: Model 2 plus HOMA-IR. PH, post-challenge hyperglycemia.
